# Measurements of the number of specified and unspecified cells in the shoot apical meristem during a plastochron in rice (*Oryza sativa*) reveal the robustness of cellular specification process in plant development

**DOI:** 10.1371/journal.pone.0269374

**Published:** 2022-06-03

**Authors:** Misuzu Nosaka-Takahashi, Makio Kato, Toshihiro Kumamaru, Yutaka Sato

**Affiliations:** 1 National Institute of Genetics, Shizuoka, Japan; 2 Department of Genetics, School of Life Science, Sokendai (Graduate University for Advanced Studies), Shizuoka, Japan; 3 Graduate School of Bioagricultural Sciences, Nagoya University, Aichi, Japan; 4 Faculty of Agriculture, Institute of Genetic Resources, Kyushu University, Fukuoka, Japan; Leiden University Faculty of Science: Universiteit Leiden Faculteit der Wiskunde en Natuurwetenschappen, NETHERLANDS

## Abstract

The shoot apical meristem (SAM) is composed of a population of stem cells giving rise to the aboveground parts of plants. It maintains itself by controlling the balance of cell proliferation and specification. Although knowledge of the mechanisms maintaining the SAM has been accumulating, the processes of cellular specification to form leaves and replenishment of unspecified cells in the SAM during a plastochron (the time interval between which two successive leaf primordia are formed) is still obscure. In this study, we developed a method to quantify the number of specified and unspecified cells in the SAM and used it to elucidate the dynamics of cellular specification in the SAM during a plastochron in rice. *OSH1* is a *KNOX* (*KNOTTED1-like homeobox*) gene in rice that is expressed in the unspecified cells in the SAM, but not in specified cells. Thus, we could visualize and count the nuclei of unspecified cells by fluorescent immunohistochemical staining with an anti-OSH1 antibody followed by fluorescein isothiocyanate detection. By double-staining with propidium iodide (which stains all nuclei) and then overlaying the images, we could also detect and count the specified cells. By using these measurements in combination with morphological observation, we defined four developmental stages of SAM that portray cellular specification and replenishment of unspecified cells in the SAM during a plastochron. In addition, through the analysis of mutant lines with altered size and shape of the SAM, we found that the number of specified cells destined to form a leaf primordium is not affected by mild perturbations of meristem size and shape. Our study highlights the dynamism and flexibility in stem cell maintenance in the SAM during a plastochron and the robustness of plant development.

## Introduction

The shoot apical meristem (SAM) repeatedly produces lateral organs that form the aboveground parts of plants. This repeated organ formation relies on the organization of the SAM [1,2], which is divided into several parts according to the cytohistological features of the cells. The central zone (CZ) is located at the center of the SAM and consists of stem cells and cells dividing relatively rarely. The cells in the peripheral zone (PZ), the region around the CZ, divide actively, and lateral organs, such as leaf primordia, are formed in this region. Some of the unspecified cells in the PZ are used to form differentiated lateral organs, after which the pool of unspecified cells in PZ is replenished. Such cytological organization of the SAM is sometimes readily evident as in the case of *Arabidopsis*, but sometimes less obvious, as in the case of grass species [3]; however, the basic concepts of forming lateral organs and maintenance of the SAM are believed to be common to many plant species. Repeated organ formation and maintenance of SAM are managed through a balance between cell specification and replenishment of unspecified cells. This process enables indeterminate growth in plants and is the major role of the SAM.

Many genes are involved in the organization and function of SAM. A representative mechanism that maintains SAM is mediated by signaling between *WUSCHEL-* (*WUS-*) and *CLAVATA3-* (*CLV3*-) expressing cells [4]. *WUS* is expressed in cells beneath the stem cells and promotes stem cell identity through an unknown signaling mechanism. *CLV* genes control the size and shape of SAM by suppressing *WUS* expression and restricting overproliferation of unspecified stem cells [5–8]. It is proposed that the function of the SAM is maintained by a regulatory feedback loop, in which *CLV3* suppresses *WUS* and *WUS* activates *CLV3* [9–12]. Perturbation of the homeostasis of the SAM results in either the reduction of the stem cell population in the SAM or the enlargement of SAM caused by overproliferation of unspecified stem cells [13].

In the initial step of leaf primordium formation, a population of leaf founder cells (P0) is specified at the flank of the SAM. The specified P0 cells are marked by the down-regulation of *KNOTTED1-like homeobox* (*KNOX*) genes, which are expressed in unspecified meristematic cells [14]. The site of P0 specification is associated with the local accumulation of auxin [15,16]. The position of P0 (i.e., the phyllotactic pattern) is assumed to be affected by mechanical tension produced by cell proliferation in the epidermal layer of the SAM as hypothesized by several studies [17–20]. In addition, surgical experiments suggest that a substance inhibitory to leaf initiation emanates from preexisting leaf primordia, so that the new leaf initiates at the farthest position from pre-existing primordia [21,22]. Thus, local auxin accumulation is assumed to be a readout of the complex signaling that determines the position of P0 initiation.

The pattern of P0 initiation is affected by perturbations of SAM geometry. There are several mutants in maize (*Zea mays*) and rice (*Oryza sativa*), where SAM size and shape are abnormal leading to a change in the phyllotactic pattern [23–25]. In the maize *abphyl1* (*abph1*) and rice *decussate1* (*dec1*) mutants, two leaves are formed from a single node instead of a single leaf, as in the wild type, changing the phyllotactic pattern from alternate to decussate. This change is associated with a larger, flattened SAM, possibly because of altered cytokinin response in these mutants.

Although knowledge on the relation between SAM geometry and positioning of lateral organs is increasing, it is still unknown how the size and shape of the SAM change during a plastochron (the time interval between which two successive leaf primordia are formed) and whether the SAM perturbation affects the number of cells specified for a future leaf (i.e., the size of the leaf founder cell population). Here, we analyzed the dynamics of cell specification at the SAM in rice. It is easy to predict the position of the P0 cell population in rice since its phyllotaxy is alternate distichous (i.e., one leaf per node, forming two rows on opposite sides of the stem). To distinguish unspecified and specified cells in the SAM, we used expression of *OSH1* as a molecular marker [26]. *OSH1* is a rice ortholog of *KNOTTED1* in maize. Unspecified cells accumulate OSH1 protein in their nuclei, while specified cells do not. Thus, we could measure the numbers of unspecified and specified cells in the SAM during a plastochron by counting the numbers of nuclei with and without OSH1 accumulation detected by immunohistochemical staining. We found that the number of unspecified cells in SAM oscillates during a plastochron owing to the reduction in their number caused by P0 specification, and that mild perturbations of SAM size and shape induced by mutations do not change the number of leaf founder cells, showing robustness of lateral organ formation in plants.

## Materials and methods

### Plant materials and growth conditions

Three mutant strains with abnormally shaped SAM—CM761, CM829, and CM873—were used in this study. They were identified by screening mutants with altered SAM shape among a collection of morphological mutants of *O*. *sativa* ‘Kimmaze’ mutagenized with *N*-methyl-*N*-nitrosourea (MNU). They were obtained from the National BioResource Project (NBRP)-RICE (https://shigen.nig.ac.jp/rice/oryzabase/locale/change?lang=en).

Dehusked rice seeds were collected into a 50-mL tube for sterilization. The seeds were rinsed once with sterilized water, held for 2 min in 35 mL of 70% ethanol, rinsed twice with sterilized water, held for 25 min in 35 mL of 2% sodium hypochlorite, and rinsed five times with sterilized water inside a clean bench. The sterilized seeds were germinated aseptically on Murashige and Skoog medium [27] supplemented with 3% sucrose, 0.01% *myo*-inositol, and 0.3% gellan gum at pH 5.8 in a plant tissue culture box at 29.5°C for 8 days. Eight-day-old seedlings were used for sectioning and analysis as described below. For observation of SAM by scanning electron microscopy, seedlings were transplanted into pots and grown for an additional 2 weeks before sampling. For phenotypic characterization of mutant strains, seedlings of mutant strains and the wild type were grown in a paddy field.

### Preparation of OSH1 antibody

Polyclonal antisera that recognize OSH1 protein were raised against a bacterially expressed histidine-tagged OSH1 fusion protein. The fusion protein was solubilized in a buffer containing urea (20 mM Tris-HCl pH 8.0, 100 mM NaCl, 8 M urea) and then purified with TALON Affinity Resin (Takara Bio). The purified histidine-tagged OSH1 fusion protein was used to raise OSH1 polyclonal antisera in rabbits. IgG that recognizes OSH1 protein was purified by using histidine-tagged OSH1 fusion protein blotted onto PVDF membrane (Immobilon-P, filter type: PVDF, pore size: 0.45 μm; Millipore) followed by washing, elution (0.1 M glycine-HCl, pH 3.0, 0.15 M NaCl), and neutralization (0.5 M HEPES-KOH, pH 8.5).

### Immunohistochemical staining of SAM using anti-OSH1 IgG

Five-millimeter sections of the basal part of the shoots of 8-day-old seedlings were collected and fixed in glacial acetic acid: formalin: sterilized water: ethanol (1:2:7:10 [v/v/v/v]), dehydrated, embedded in Paraplast Plus (Oxford Labware), and sectioned to 8-μm thick on a rotary microtome. The slides with sections were rehydrated, washed with sterilized water, PBS buffer, and then incubated with 0.1 μg/mL Proteinase K in PBS buffer. After washing with PBS buffer, the sections were covered with 400 μL of blocking solution (1% BSA and 0.05% Tween 20 in PBS buffer) for 30 min at 25°C. The blocking solution was then removed and the sections on each slide were covered with 50 μL of rabbit anti-OSH1 IgG diluted 1:5 in blocking solution, covered with a cover glass, and incubated overnight at 25°C. After washing, the sections were covered with 100 μL of secondary antibody (goat anti-rabbit Ig(H+L) fluorescein isothiocyanate (FITC) conjugate; Zymed 81–6111) diluted 1:600 in blocking solution, and incubated for 2 h at 25°C. After washing, the sections were covered with 600 μL of 5 μg/mL RNase A in PBS buffer and incubated for 5 min at 25°C. Then 600 μL of 0.1 μg/mL propidium iodide (PI) in PBS buffer was added to the sections, followed by incubation for 5 min at 25°C. The slides were washed twice with PBS buffer for 5 min each, and mounted with Vectashield (Vector Laboratories).

### Measuring numbers of total cells, OSH1-positive cells, and OSH1-negative cells in the SAM during a plastochron

The FITC and PI double-stained serial cross sections of SAM during a plastochron (the time interval between which two successive leaf primordia are formed) were observed through a confocal laser microscope (FV1000, Olympus). The nuclei of OSH1-positive cells were stained with FITC, and the nuclei of all cells at the shoot apex (regardless of differentiation status) were stained with PI. In each section observed, the numbers of unspecified and specified cells were obtained in FV10-ASW v. 1.4 Viewer software (Olympus). The total number of cells in a SAM was calculated as the sum.

### Measuring the size and shape of SAM

**Fig 1:**
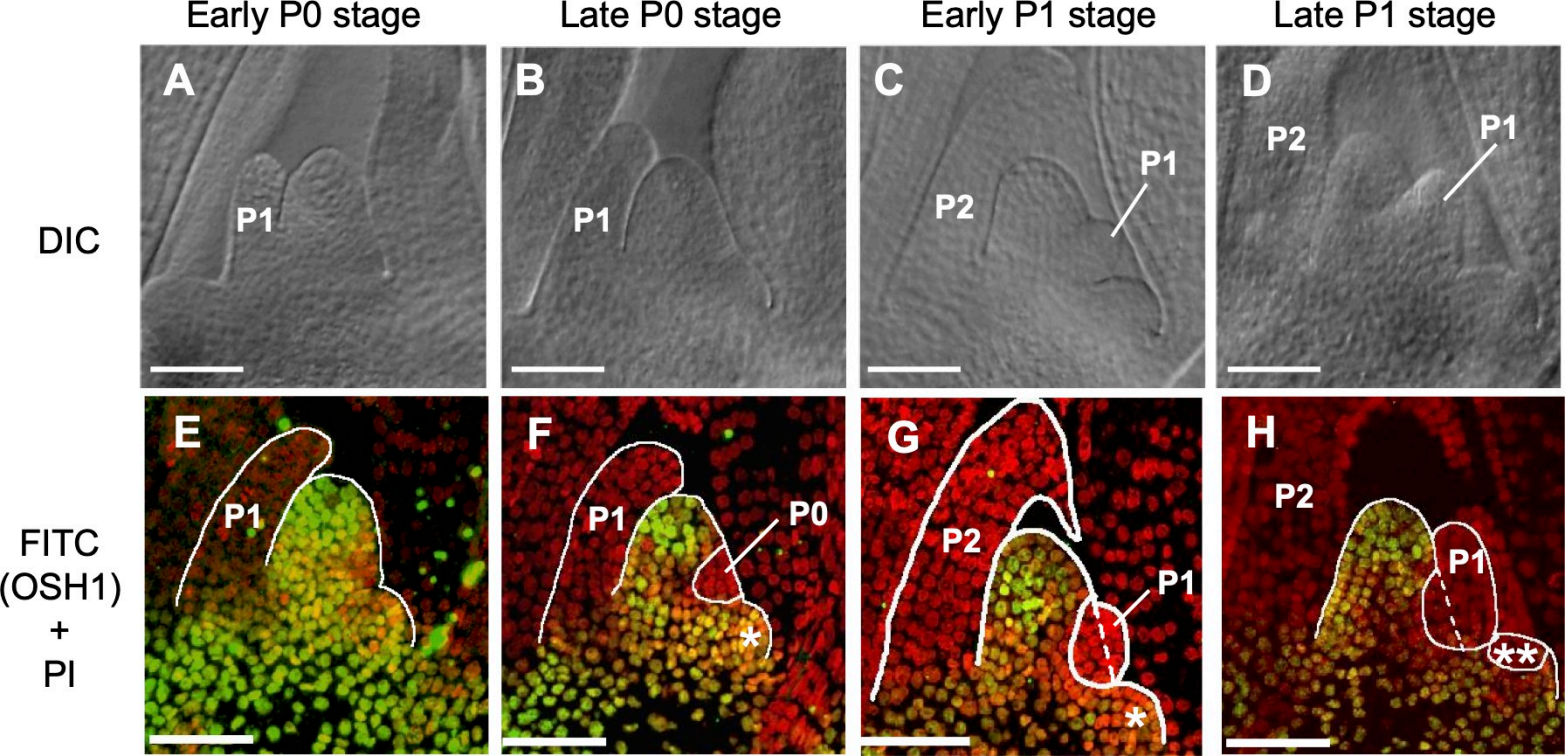
Dynamic transformation of morphology and OSH1 accumulation pattern in shoot apical meristem during a plastochron in rice. (A–D) Differential interference-contrast images of a SAM during a plastochron in wild type rice. (E–H) FITC (OSH1) and PI double staining of longitudinal sections of a SAM during a plastochron. Red signals indicate PI-stained nuclei. Green signals indicate OSH1 localization visualized by anti-OSH1 antibody and FITC. PI signals and OSH1 signals were captured independently and overlaid. P0, P1, and P2 indicate the stage of leaf development [29]. Labels at the top indicate the four stages within a plastochron that we defined. (A, E) Early P0 stage. (B, F) Late P0 stage. (C, G) Early P1 stage. (D, H) Late P1 stage. (*) Leaf base. (**) Leaf edge. Dashed line indicates the border of meristem and bulge of P1. Scale bars: 50 μm.

Longitudinal sections including SAM were collected from the basal part of the shoot of 8-day-old seedlings with a razor knife. The sections were fixed in glacial acetic acid: formalin: sterilized water: ethanol (1:2:7:10 [v/v/v/v]), and then dehydrated in a graded ethanol series. After tissue clearing through a graded series of methyl salicylate: ethanol (1:2, 1:1, 2:1, 1:0, 1:0 [v/v]), we observed the shoot apices through a microscope equipped with Nomarski differential interference-contrast optics (Olympus). SAMs at the early P1 stage (defined in Fig 1) were used for measurement of width, height, shape, and volume. The width of the SAM was defined as the length of the baseline set at the point where P1 was attached to the SAM. To set the baseline of the SAM continuously at the point where leaf primordium was attached during a plastochron, baseline of the SAM was set at the point where P2 was attached to the SAM at early P1 and late P1 stages since P1 was counted as P2 at these later stages. The height was defined as the length of a perpendicular line drawn from the peak of the shoot apex to the baseline. The shape was measured as the height/width quotient. The volume was approximated by a paraboloid [28].

### Scanning electron microscopy

Shoot apices of wild type and mutant seedlings were collected with the aid of a stereomicroscope. They were fixed in glacial acetic acid: formalin: sterilized water: ethanol (1:2:7:10 [v/v/v/v]) and dehydrated through a graded ethanol series. After replacement of the solvent with acetone, samples were dried to their critical point in a Critical Point Dryer (Hitachi), sputter-coated with platinum in an E-1030 Ion Sputter Coater (Hitachi), and observed through a scanning electron microscope (S-3000N, Hitachi).

### *In situ* hybridization

Shoot apices were fixed and embedded as for the immunohistochemical analysis. Sections were cut 8 μm thick on a rotary microtome. *In situ* hybridization was performed as described previously [30] at 55°C overnight. To produce DIG-labeled *FON2* and Histone *H4* sense and antisense probes, full-length *FON2* and Histone *H4* cDNAs of rice without the poly-A region were amplified, purified, and used as templates for *in vitro* transcription (1 μg template, 2 μL NTP lab mix, 0.5 μL RNase inhibitor [Takara Bio], 0.1 μL 0.1 M DTT, 4 μL 5× Buffer [Stratagene], 1 μL T7 RNA polymerase [Stratagene]).

## Results

### Stages of leaf initiation at SAM

Since the morphology of the SAM changes during a plastochron, we divided the leaf initiation step at the SAM into four stages: early P0 (Fig 1A), late P0 (Fig 1B), early P1 (Fig 1C), and late P1 (Fig 1D) stages. At the early P0 and late P0 stages (Fig 1A and 1B), the pre-existing P1 leaf primordium formed a hood-like structure surrounding the SAM. The early P0 and late P0 stages are difficult to distinguish morphologically; however, the OSH1 expression clearly distinguished these stages (Fig 1E and 1F). While cells at the SAM are still OSH1-positive at the early P0 stage, OSH1-negtive cells appear at the subsequent stages. Consequently, the region consisted of OSH1-negative cells indicates the region specified to form a leaf at the SAM. At the late P0 stage, though not at the early P0 stage, a population of P0 cells was visible opposite the P1 leaf primordium (Fig 1E and 1F). As cells in the P0 region proliferate and grow (cell expansion), a bulge is formed at the flank of the SAM; hereafter, P0 becomes P1, while the previous P1 becomes P2. At the early P1 stage, the P1 leaf primordium became clearly visible as a bulge (Fig 1C and 1G). At the late P1 stage, the base of the P1 leaf primordium started to encircle the SAM (Fig 1D and 1H). At this point, the early P0 stage of the next plastochron starts. Thus, we found that, during a plastochron, geometry of the site of leaf initiation (P0-P1) is changing in the SAM and the numbers of unspecified and specified cells in the SAM visualized by OSH1 immunostaining also change dynamically.

### Numbers of unspecified and specified cells in the SAM during a plastochron

**Fig 2:**
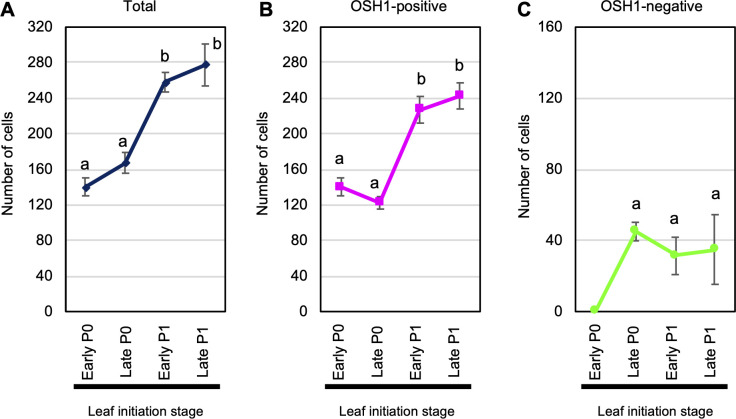
Transitions in numbers of total cells, OSH1-positive cells, and OSH1-negative cells in wild type shoot apical meristem during a plastochron. Through OSH1 immunostaining, we measured the numbers of (A) total cells (blue line), (B) OSH1-positive cells (pink line, indicating unspecified cells), and (C) OSH1-negative cells (green line, indicating specified cells) in the SAM. The *x*-axis indicates the leaf initiation stage at the SAM: early P0, late P0, early P1, and late P1 stages (see Fig 1). The *y*-axis indicates the number of cells. Data are means; error bars indicate s.d. Number of samples (meristems) measured: early P0 stage (*n* = 3), late P0 stage (*n* = 3), early P1 stage (*n* = 6), late P1 stage (*n* = 7). Different letters above the error bars represents significant differences of number of cells among the stages by Tukey-Kramer’s test (*P* < 0.01).

To quantitatively describe the process of P0 specification and replenishment of unspecified cells in the SAM during a plastochron, we separately counted the OSH1-immunostaining-positive and -negative nuclei in serial cross sections of SAM (Figs 2 and S1–S4). We set the base of the SAM region at the lowest point where the SAM is attached to P1 at the early P0 and late P0 stages, or at the lowest point where it is attached to P2 at the early P1 and late P1 stages since P1 was counted as P2 at these stages. Using this definition, we summed the numbers of OSH1-positive and -negative nuclei in all sections above the base of the SAM to obtain the total for each cell type and then added these together to obtain the total number of cells within the meristem.

The numbers of total cells, unspecified cells, and specified cells in a SAM during a plastochron are plotted in Fig 2. The total number of cells in the SAM increased slightly during the transition from early P0 to late P0, increased dramatically during the transition from late P0 to early P1, and increased slightly during the transition from early P1 to late P1 stage (Fig 2A). The number of unspecified (OSH1-positive) cells in the SAM decreased slightly during the transition from early P0 to late P0, then increased until the late P1 stage (Fig 2B). A population of specified cells (i.e., OSH1-negative cells) appeared at the late P0 stage. The number of cells specified to leaf primordium within the dome at SAM did not change significantly after the late P0 stage, although the domain of OSH1-negative cells broadened laterally at the base of the SAM (Figs 2C and S4R).

### Mutant rice strains with altered SAM size and shape

**Fig 3:**
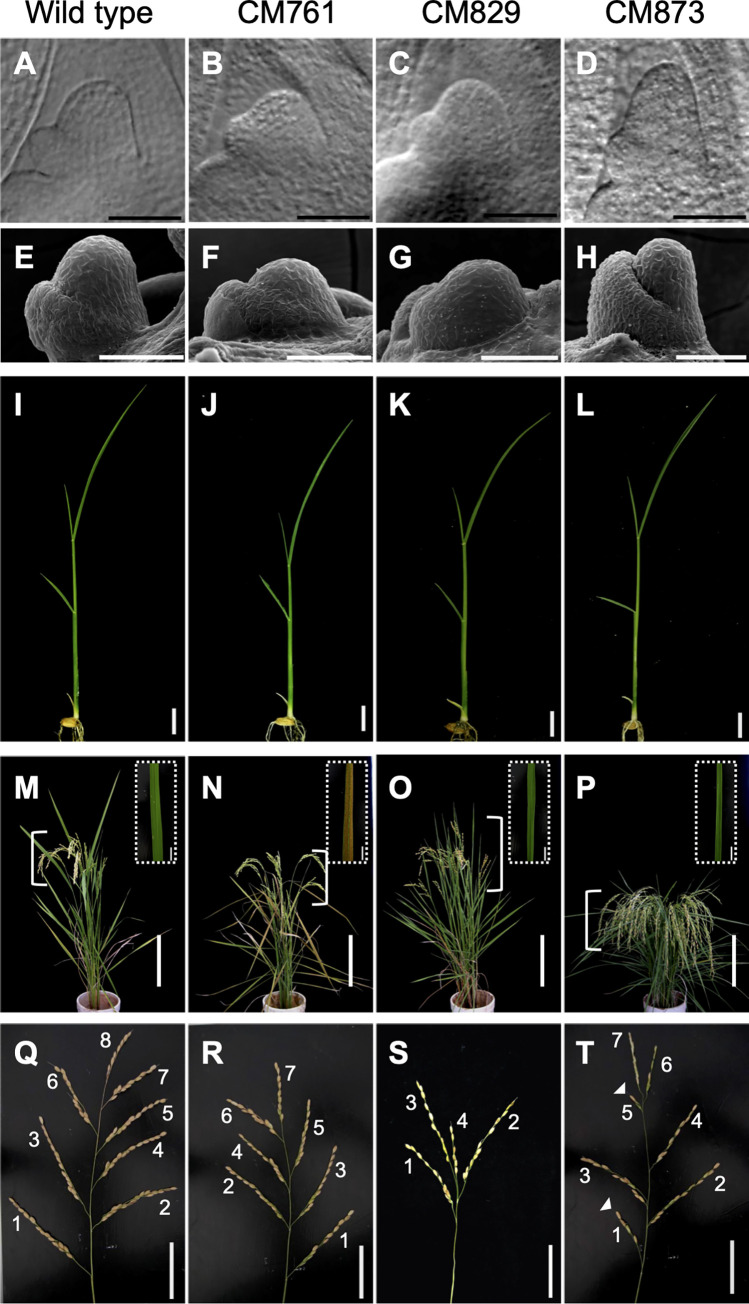
Selected mutants of shoot apical meristem with altered size and shape. (A–D) Morphology of the SAM of 8-day-old (A) wild type and (B–D) mutant plants at the early P1 stage observed through a microscope equipped with Nomarski differential interference-contrast optics. (E–H) Morphology of the SAM of 3-week-old (E) wild type and (F–H) mutant plants observed through SEM. (I–L) Morphology of the shoot of 8-day-old (I) wild type and (J–L) mutant plants. (M–P) Aboveground plant parts of (M) wild type and (N–P) mutants grown for 4 months after germination. Position of panicles are marked with braces. Insets indicate magnification of a flag leaf from each plant. (Q–T) Panicle architecture of (Q) wild type and (R–T) mutants. Number of branching are labelled and shortened branches are indicated by arrowheads. (A, E, I, M, Q) wild type; (B, F, J, N, R) CM761; (C, G, K, O, S) CM829; (D, H, L, P, T) CM873. Scale bars: 50 μm in A–H; 1 cm in I–L; 20 cm in M–P; 1 cm in M–P insets; 5 cm in Q–T.

Perturbation of the size and shape of the SAM is a direct approach to understanding the mechanisms of replenishment of unspecified cells and of cell specification in the SAM and their robustness. To take this approach, we used three mutant strains, CM761, CM829, and CM873. The SAMs of CM761 and CM829 are smaller and flatter and that of CM873 is slimmer than that of the wild type (Table 1, Fig 3A–3H). Although we could observe morphological abnormalities in the SAM of 8-day-old seedlings of the three mutant strains, none of them showed obvious gross morphological phenotypes at that stage (Fig 3I–3L). Later in development, however, the mutants showed various abnormalities, including short stature (CM761 and CM873), more frequent tillering (CM829 and CM873), reduced and shortened panicle branching in CM829 and CM873, respectively (Fig 3I–3T). These morphological abnormalities observed only at later developmental stages suggest that these mutants have a defect in proper organization of SAM from early developmental stage, while they continue development until maturity. Since abnormalities in SAM organization is known to induce abnormal development of organs derived from SAM [28], such as stems, axils, and branches of panicles, the various abnormalities observed in mutants are assumed to be induced by abnormal organization of the SAM in the mutants.

**Table 1:** Width, height, shape, and volume of shoot apical meristem of wild type and mutants at early P1 stage.

Strain	Width (μm)^a^	Height (μm)^a^	Shape (Height/Width)^a^	Volume (× 10^4^ μm^3^)^a^	*n* ^ ^ ^b^
Wild type	70.5 ± 5.1	62.3 ± 5.3	0.88 ± 0.06	12.2 ± 2.7	7
CM761	63.7 ± 2.6*	40.8 ± 2.0**	0.64 ± 0.03**	6.53 ± 0.8**	5
CM829	60.2 ± 4.1**	43.7 ± 4.6**	0.73 ± 0.05**	6.29 ± 1.4**	4
CM873	62.9 ± 3.3**	68.1 ± 5.2*	1.08 ± 0.07**	10.6 ± 1.8	9

^a^ Mean ± s.d.

^b^ *n* indicates the number of SAMs measured at each stage.

Asterisks indicate significant differences from wild type (Student’s *t*-test; **P < 0.01, *P < 0.05).

**Fig 4:**
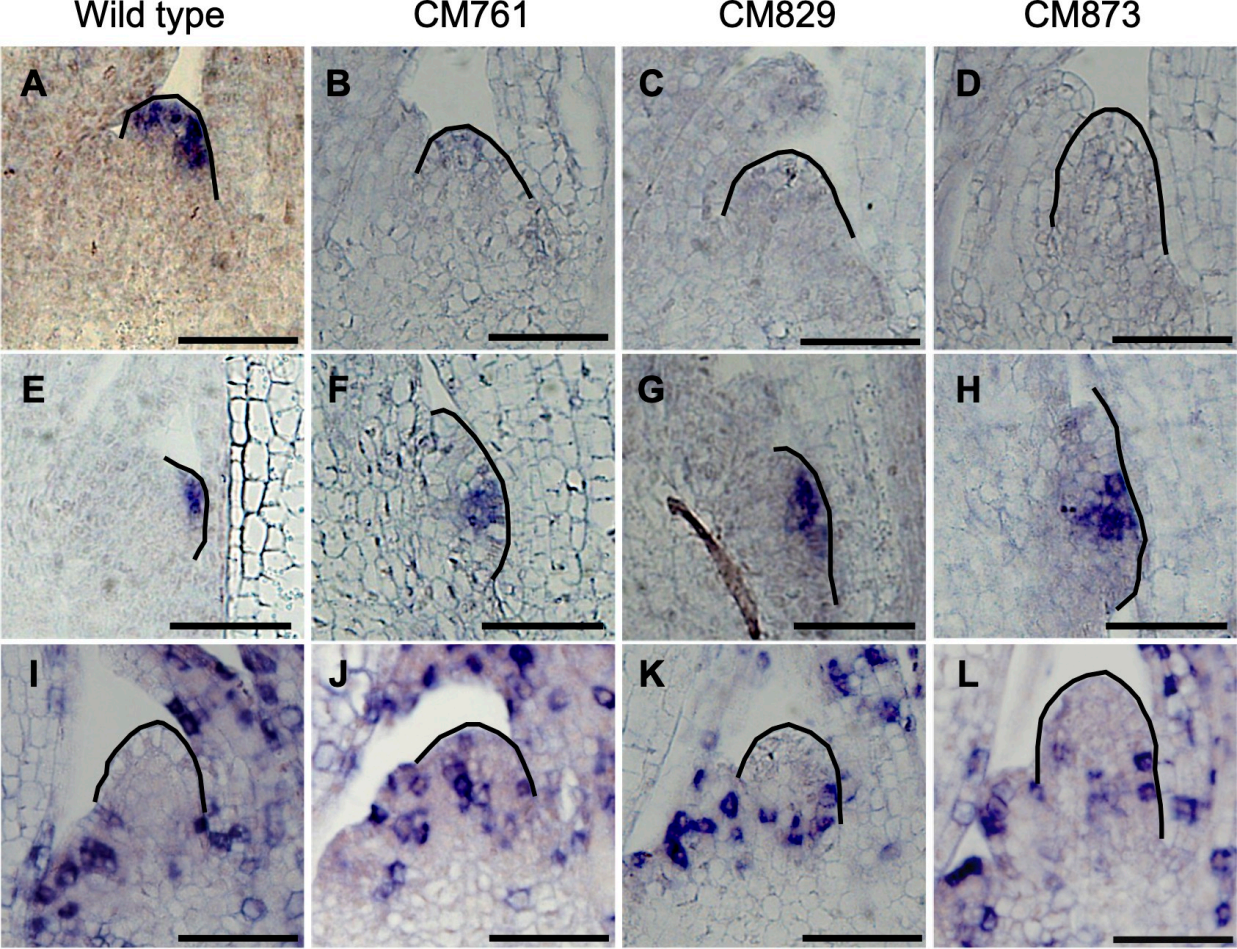
*FON2* and histone *H4* expression in meristems of wild type and mutants. (A–H) *FON2* expression (A–D) in SAM and (E–H) axillary meristem in the early vegetative phase of (A, E) wild type, (B, F) CM761, (C, G) CM829, and (D, H) CM873. (I–L) Histone *H4* expression in SAM in the early vegetative phase of (I) wild type, (J) CM761, (K) CM829, and (L) CM873. Meristems are outlined. Scale bars: 50 μm.

Consistent with the morphological abnormalities observed in the SAM of mutants, expression of one of the stem cell markers in SAM, *FON2*, which encodes one member of the CLE peptide family in rice [31], became abnormal in these mutants (Fig 4A–4D). In CM761, *FON2* expression in the SAM was weaker than in the wild type, and in CM829 and CM873, it was hardly detectable. In contrast, *FON2* expression in developing axillary meristem at the vegetative stage in all three mutant strains was similar to that in the wild type (Fig 4E–4H). This observation provides further evidence that the defects in these three mutant strains occur in the maintenance of SAM. We further analyzed the molecular phenotype of these mutant strains by analyzing the expression of histone *H4* in the SAM (Fig 4I–4L). Since histone *H4* gene is specifically expressed in the S phase of the cell cycle, it is a good marker of cell division activity. Cells expressing histone *H4* were rarely seen in the SAM of the wild type but more often observed in mutants, especially in CM761. This indicates that the meristem of the mutant lines has higher cell division activity compared to that of wild type to maintain the meristem homeostasis. As the next step, we used these mutants to ask how mild perturbation of homeostasis of shoot apical meristem affects P0 specification and replenishment of unspecified cells.

### Numbers of unspecified and specified cells in mutants with perturbed size and shape of SAM

To analyze the numbers of unspecified and specified cells in the SAM when its size and shape are altered, we conducted OSH1 immunostaining using the SAMs of the three mutant strains at the late P0 and early P1 stages, and measured the numbers of OSH1-positive and -negative cells as in the wild type (S5–S10 Figs). We did not measure the numbers of OSH1-positive and -negative cells at early P0 and late P1 stages since the number of OSH1-negative cells is zero at early P0 stage and late P1 stage is the timing after the cell specification. In all three mutant lines, the plants at 8 days after germination emerged 4th leaf and developed 7th leaf primordium as the same timepoint as the wild type. Thus, we concluded that we could compare the numbers of specified and unspecified cells in the SAM of wild type and mutants at the same developmental stage.

**Fig 5:**
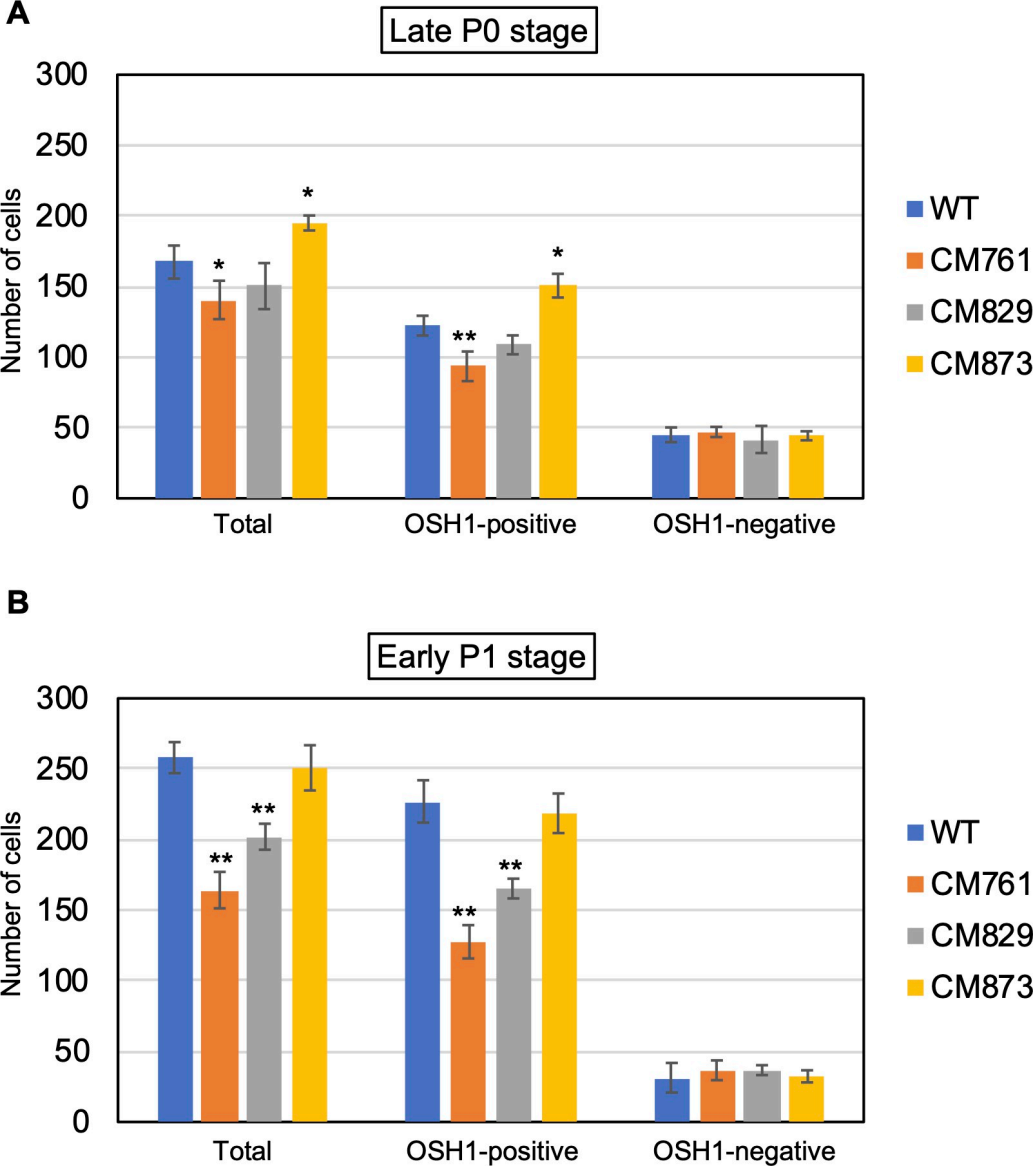
Numbers of total cells, OSH1*-*positive cells, and OSH1-negative cells in the shoot apical meristem of wild type and mutants at the late P0 and early P1 stages during a plastochron. Numbers of cells were compared among wild type (blue bars), CM761 (orange bars), CM829 (gray bars), and CM873 (yellow bars) at the (A) late P0 and (B) early P1 stages. The *x*-axis indicates the type of cell; the *y*-axis indicates the number of cells. Data are means; error bars indicate s.d. Asterisks indicate significant differences from wild type (Student’s *t*-test; **P < 0.01, *P < 0.05). Numbers of samples (meristems) measured: wild type (*n* = 3), CM761 (*n* = 4), CM829 (*n* = 3), CM873 (*n* = 3) at late P0 stage; wild type (*n* = 6), CM761 (*n* = 9), CM829 (*n* = 7), CM873 (*n* = 8) at early P1 stage.

The numbers of OSH1-positive and -negative cells in the SAM at the late P0 and early P1 stages of the three mutant strains and the wild type are shown in Fig 5. In CM761 with reduced height and flattened shaped SAM at early P1 stage, the numbers of total and unspecified cells in the SAM at both late P0 stage and early P1 stage were significantly reduced compared to the wild type (Table 1, Figs 5, S5 and S6). In CM829 with reduced height and flattened shaped SAM at early P1 stage, the numbers of total and unspecified cells in the SAM did not change significantly at late P0 stage but they were significantly reduced at the early P1 stage compared to the wild type, (Table 1, Figs 5, S7 and S8). In CM873 with slender and slightly tall shaped SAM of similar volume with wild type at early P1 stage, the numbers of total and unspecified cells in the SAM were significantly increased at late P0 stage but they did not change significantly at the early P1 stage compared to the wild type, (Table 1, Figs 5, S9 and S10). In contrast to the numbers of total and unspecified cells, the number of OSH1-negative (i.e., specified) cells in SAM among the three mutants did not differ significantly from the wild type at either stage (Fig 5). These results indicate that the number of unspecified cells in the SAM varies depending on its size and shape, but that the number of specified cells contributing to P0 specification stays constant.

## Discussion

Homeostasis of the SAM is an important mechanism that enables long-lasting plant development. The SAM repeatedly produces lateral organs such as leaves through specification of the P0 (leaf founder) cells within it. The cells used to form P0 are replenished before the meristem produces another P0. Thus, the balance between consumption and replenishment enables the plant to grow continuously. Genetic perturbation of this balance has contributed to understanding the mechanisms of homeostasis during vegetative and reproductive stages of development [4]; however, information on the process of cellular specification and replenishment of unspecified cells during the formation of two successive leaves within a SAM—i.e., during a plastochron—has been limited.

We tackled this issue by morphological observation of SAMs in rice and by direct measurement of the numbers of unspecified and specified cells in the SAM during a plastochron. To quantitatively describe the dynamics of the SAM, we defined four developmental stages based on morphology and cellular differentiation. The SAMs collected from 8-day-old seedlings included all four developmental stages, early P0, late P0, early P1, and, late P1. This could be caused by slight differences in the timing of germination, because plastochron is usually constant among individuals [32–34]. Although the early P0 and late P0 stages are difficult to distinguish by their morphology, OSH1 immunostaining clearly distinguishes them by the absence or presence, respectively, of a population of OSH1-negative (i.e., specified) cells (Fig 1E and 1F), which are especially visible in cross sections (S1 and S2 Figs). This suggests that a population of around 45 cells in the SAM are specified within a relatively short period of time. The determination of P0 position is controlled by local auxin accumulation. Mechanical stress which could be produced by auxin-induced cell growth is a candidate of the trigger of leaf initiation [20], and it might affect cells in P0 domain so that a population of around 45 cells in the SAM can be specified almost synchronously. The dynamic changes in the number of unspecified and specified cells in the SAM suggest that there must be an elaborate mechanism to balance the consumption and replenishment of unspecified cells in the SAM for multiple rounds of cycles of leaf initiation.

Indeed, genetic perturbation of such homeostasis of SAM by mutations results in smaller or larger SAMs [13]. Mutations in the CLV pathway result in larger meristems often having a flattened shape, sometimes accompanied by a bifurcated or fasciated stem [5,6,35–38]. On the other hand, there are also mutations that produce smaller meristems such as *wus* in *Arabidopsis* and *kn1* in maize [14,39–42]. The phenotypes of those mutations have been described in vegetative, inflorescence, or floral meristems, but there is almost no information on the dynamics of cellular differentiation of those mutants during a plastochron. Here, we developed a method to analyze the cellular specification in the SAM quantitatively during a plastochron. This enabled us to conduct unique analysis on how perturbation of the size and shape of the SAM affects the number of unspecified and specified cells in newly screened mutant lines.

We selected three mutant lines with altered shape and size of the SAM. The aboveground part of these mutants showed no obvious differences from the wild type at 8 days after germination, although the size and shape of the SAM became malformed in all three mutants. This is striking because many of the previously described mutations that affect the size and shape of the SAM at the vegetative stage have major effects on gross plant morphology. In maize *abphyl1* and rice *dec1* mutant plants, the SAM is larger and flatter than in the wild type. Both mutants show a transition of the phyllotactic pattern from distichous to decussate and bear narrower leaves than the wild type. A series of mutations that affect plastochron length in rice and barley, such as *pla1*, *pla2*, *pla3*, *mnd1*, *mnd4*, and *mnd8*, also have defects in meristem size, with abnormal plant stature and leaf shape [32–34,43]. As all three mutant lines isolated in this study showed relatively mild meristem phenotypes without discernable morphological defects at 8 days old, we suggest that there is a mechanism that compensates for relatively weak meristem abnormalities and to continue development.

Despite the mild phenotypes in these mutants in the size and shape of the SAM, the expression of a molecular maker for the stem cell region of the SAM in rice, *FON2*, clearly revealed meristem defects in all three mutants. Interestingly, the expression of *FON2* in the incipient axillary meristems of all three mutants was indistinguishable from that in the wild type. This suggests that the expression of *FON2* is sensitive to perturbation in the homeostasis of SAM. Indeed, *FON2* itself is not required for controlling the size of SAM during the vegetative stage, although it is expressed in the stem cell region of the SAM at that stage [44]. On the other hand, simultaneous inducible knockdown of *FCP1* and *FCP2*, which encode FON2-like CLE proteins, affected the vegetative SAM and resulted in an enlarged *FON2* expression domain [44,45]. This suggests that defects in the three mutants we examined are not in the FCP1/2-mediated CLV-like signaling pathway. Thus, yet-unknown pathways must maintain meristem homeostasis.

The three mutant lines were selected thorough the following two criteria; 1) The mutant shows no morphological deficiencies during the 8-day-old seedling stage, but at the later developmental stage, pleiotropic morphological deficiencies are observed. 2) The mutant SAM shows mild morphological deficiencies in its size and shape. Thus, these mutant lines are ideal materials to analyze the effects of perturbed size and shape of SAM at the 8-day-old seedling stage. On the other hand, the relationship between the morphological deficiencies observed in matured plant and the perturbed size and shape of SAM at the seedling stage remains to be answered. The deficiencies in the mutant SAM size and shape may increase severity progressively during development. While the mutants are able to continue development without morphological deficiencies to some extent, in the late developmental stage, when the defects in SAM organization go over the acceptable capacity of the SAM, the mutants may show defects in morphology. In future, the relationship between defects of SAM organization and morphological deficiencies observed at later developmental stage in the mutants will be clarified through comparing phenotypes observed in SAM of wild type and mutants in each developmental stage, and observing the expression of marker genes expressed in the SAM at cell specification stage during the formation of leaf primordium, stems, including nodes and internodes, axils, and branches of inflorescence after developing these marker genes.

In summary, we clarified the dynamic transition of numbers of unspecified and specified cells in the SAM at the shoot apex during a plastochron in rice. In addition, by using mutant lines with relatively mild perturbation of the size and shape of the SAM, we showed that the cell specification process giving rise to leaf initiation is robust against such perturbations. We propose that this characteristic is important to stably continue normal development in a fluctuating environment. Identification of molecular lesions in the three mutant lines will provide further information on the regulation of the homeostasis of the SAM.

## Supporting information

S1 Fig OSH1-immunostained shoot apical meristem of wild type at early P0 stage. (A) A longitudinal section and (B–M) serial cross sections of the wild type SAM at early P0 stage. (B, F, J) Merged images of FITC signals, PI signals, and differential interference-contrast (DIC) images. (C, G, K) Green signals indicate nuclei of OSH1-positive cells visualized by anti-OSH1 antibody and FITC. (D, H, L) Red signals indicate PI-stained nuclei of whole cells. (E, I, M) DIC images. Each set of cross-section images (B–E, F–I, and J–M) is linked to the corresponding part of the longitudinal section (A) by a blue line. White dashed lines in the longitudinal section (A) indicate the borders of the cross sections (B–M). (B, E, F, I, J, M) The circled region in each panel indicates the SAM. (A, B, E, F, I, J, M) White arrowheads indicate P1. Scale bars: 50 μm.
(PDF)

S2 Fig OSH1-immunostained shoot apical meristem of wild type at late P0 stage. (A) A longitudinal section and (B–Q) serial cross sections of the wild type SAM at late P0 stage. (B, F, J, N) Merged images of FITC signals, PI signals, and differential interference-contrast (DIC) images. (C, G, K, O) Green signals indicate nuclei of OSH1-positive cells visualized by anti-OSH1 antibody and FITC. (D, H, L, P) Red signals indicate PI-stained nuclei of whole cells. (E, I, M, Q) DIC images. Each set of cross-section images (B–E, F–I, J–M, and N–Q) is linked to the corresponding part of the longitudinal section (A) by a blue line. White dashed lines in the longitudinal section (A) indicate the borders of the cross sections (B–Q). (B, E, F, I, J, M, N, Q) The circled region in each panel indicates the SAM. (A, E, I, M, Q) White arrowheads indicate P1. Scale bars: 50 μm.
(PDF)

S3 Fig OSH1-immunostained shoot apical meristem of wild type at early P1 stage. (A) A longitudinal section and (B–U) serial cross sections of wild type SAM at early P1 stage. (B, F, J, N, R) Merged images of FITC signals, PI signals, and differential interference-contrast (DIC) images. (C, G, K, O, S) Green signals indicate nuclei of OSH1-positve cells visualized by anti-OSH1 antibody and FITC. (D, H, L, P, T) Red signals indicate PI-stained nuclei of whole cells. (E, I, M, Q, U) DIC images. Each set of cross-section images (B–E, F–I, J–M, N–Q, and R–U) is linked to the corresponding part of the longitudinal section (A) by a blue line. White dashed lines in the longitudinal section (A) indicate the borders of the cross sections (B–U). (B, E, F, I, J, M, N, Q, R, U) The circled region in each panel indicates the SAM. (A, N, Q, R, U) White arrowheads indicate P1. Scale bars: 50 μm.
(PDF)

S4 Fig OSH1-immunostained shoot apical meristem of wild type at late P1 stage. (A) A longitudinal section and (B–U) serial cross sections of the wild type SAM at late P1 stage. (B, F, J, N, R) Merged images of FITC signals, PI signals, and differential interference-contrast (DIC) images. (C, G, K, O, S) Green signals indicate nuclei of OSH1-positive cells visualized by anti-OSH1 antibody and FITC. (D, H, L, P, T) Red signals indicate PI-stained nuclei of whole cells. (E, I, M, Q, U) DIC images. Each set of cross-section images (B–E, F–I, J–M, N–Q, and R–U) is linked to the corresponding part of the longitudinal section (A) by a blue line. White dashed lines in the longitudinal section (A) indicate the borders of the cross sections (B–U). (B, E, F, I, J, M, N, Q, R, U) The circled region in each panel indicates the SAM. (A, F, I, J, M, N, Q, R) White arrowheads indicate P1. Scale bars: 50 μm.
(PDF)

S5 Fig OSH1-immunostained shoot apical meristem of CM761 at late P0 stage. (A) A longitudinal section and (B–M) serial cross sections of the SAM of CM761 at late P0 stage. (B, F, J) Merged images of FITC signals, PI signals, and differential interference-contrast (DIC) images. (C, G, K) Green signals indicate nuclei of OSH1-positive cells visualized by anti-OSH1 antibody and FITC. (D, H, L) Red signals indicate PI-stained nuclei of whole cells. (E, I, M) DIC images. Each set of cross-section images (B–E, F–I, and J–M) is linked to the corresponding part of the longitudinal section (A) by a blue line. White dashed lines in the longitudinal section (A) indicate the borders of the cross sections (B–M). (B, E, F, I, J, M) The circled region in each panel indicates the SAM. (A, E, I, M) White arrowheads indicate P1. Scale bars: 50 μm.
(PDF)

S6 Fig OSH1-immunostained shoot apical meristem of CM761 at early P1 stage. (A) A longitudinal section and (B–Q) serial cross sections of the SAM of CM761 at early P1 stage. (B, F, J, N) Merged images of FITC signals, PI signals, and differential interference-contrast (DIC) images. (C, G, K, O) Green signals indicate nuclei of OSH1-positive cells visualized by anti-OSH1 antibody and FITC. (D, H, L, P) Red signals indicate PI-stained nuclei of whole cells. (E, I, M, Q) DIC images. Each set of cross-section images (B–E, F–I, J–M, and N–Q) is linked to the corresponding part of the longitudinal section (A) by a blue line. White dashed lines in the longitudinal section (A) indicate the borders of the cross sections (B–Q). (B, E, F, I, J, M, N, Q) The circled region in each panel indicates the SAM. (A, J, M, N, Q) White arrowheads indicate P1. Scale bars: 50 μm.
(PDF)

S7 Fig OSH1-immunostained shoot apical meristem of CM829 at late P0 stage. (A) A longitudinal section and (B–M) serial cross sections of the SAM of CM829 at late P0 stage. (B, F, J) Merged images of FITC signals, PI signals, and differential interference-contrast (DIC) images. (C, G, K) Green signals indicate nuclei of OSH1-positive cells visualized by anti-OSH1 antibody and FITC. (D, H, L) Red signals indicate PI-stained nuclei of whole cells. (E, I, M) DIC images. Each set of cross-section images (B–E, F–I, J–M) is linked to the corresponding part of the longitudinal section (A) by a blue line. White dashed lines in the longitudinal section (A) indicate the borders of the cross sections (B–M). (B, E, F, I, J, M) The circled region in each panel indicates the SAM. (A, E, I, M) White arrowheads indicate P1. Scale bars: 50 μm.
(PDF)

S8 Fig OSH1-immunostained shoot apical meristem of CM829 at early P1 stage. (A) A longitudinal section and (B–Q) serial cross sections of the SAM of CM829 at early P1 stage. (B, F, J, N) Merged images of FITC signals, PI signals, and differential interference-contrast (DIC) images. (C, G, K, O) Green signals indicate nuclei of OSH1-positive cells visualized by anti-OSH1 antibody and FITC. (D, H, L, P) Red signals indicate PI-stained nuclei of whole cells. (E, I, M, Q) DIC images. Each set of cross-section images (B–E, F–I, J–M, and N–Q) is linked to the corresponding part of the longitudinal section (A) by a blue line. White dashed lines in the longitudinal section (A) indicate the border of the cross sections (B–Q). (B, E, F, I, J, M, N, Q) The circled region in each panel indicates the SAM. (A, J, M, N, Q) White arrowheads indicate P1. Scale bars: 50 μm.
(PDF)

S9 Fig OSH1-immunostained shoot apical meristem of CM873 at late P0 stage. (A) A longitudinal section and (B–U) serial cross sections of the SAM of CM873 at late P0 stage. (B, F, J, N, R) Merged images of FITC signals, PI signals, and differential interference-contrast (DIC) images. (C, G, K, O, S) Green signals indicate nuclei of OSH1-positive cells visualized by anti-OSH1 antibody and FITC. (D, H, L, P, T) Red signals indicate PI-stained nuclei of whole cells. (E, I, M, Q, U) DIC images. Each set of cross-section images (B–E, F–I, J–M, N–Q, R–U) is linked to the corresponding part of the longitudinal section (A) by a blue line. White dashed lines in the longitudinal section (A) indicate the border of the cross sections (B–U). (B, E, F, I, J, M, N, Q, R, U) The circled region in each panel indicates the SAM. (A, E, I, M, Q, U) White arrowheads indicate P1. Scale bars: 50 μm.
(PDF)

S10 Fig OSH1-immunostained shoot apical meristem of CM873 at early P1 stage. (A) A longitudinal section and (B–Y) serial cross sections of the SAM of CM873 at early P1 stage. (B, F, J, N, R, V) Merged images of FITC signals, PI signals, and differential interference-contrast (DIC) images. (C, G, K, O, S, W) Green signals indicate nuclei of OSH1-positive cells visualized by anti-OSH1 antibody and FITC. (D, H, L, P, T, X) Red signals indicate PI-stained nuclei of whole cells. (E, I, M, Q, U, Y) DIC images. Each set of cross-section images (B–E, F–I, J–M, N–Q, R–U, and V–Y) is linked to the corresponding part of the longitudinal section (A) by a blue line. White dashed lines in the longitudinal section (A) indicate the borders of the cross sections (B–Y). (B, E, F, I, J, M, N, Q, R, U, V, Y) The circled region in each panel indicates the SAM. (A, N, Q, R, U, V) White arrowheads indicate P1. Scale bars: 50 μm.
(PDF)

S11 Fig Measurement of size and shape of the shoot apical meristem at the early P1 stage. The width of the SAM was defined as the length of the baseline set at the point where P2, which was P1 in the previous stages, was attached to the SAM. The height was defined as the length of a perpendicular line drawn from the peak of the shoot apex to the baseline. The width (yellow line), height (red line), shape (height/width), and volume of the SAM were measured. P1 and P2 indicate leaf primordia. (*) indicates leaf base of P2.
(PDF)
