# Supplementary material for: Measurements of the number of specified and unspecified cells in the shoot apical meristem during a plastochron in rice (Oryza sativa) reveal the robustness of cellular specification process in plant development
Source: PLoS One. 2022 Jun 3;17(6):e0269374. doi: 10.1371/journal.pone.0269374 (PMC9165865; doi:10.1371/journal.pone.0269374)
Supplement: S1 Fig — (A) A longitudinal section and (B–M) serial cross sections of the wild type SAM at early P0 stage. (B, F, J) Merged images of FITC signals, PI signals, and differential interference-contrast (DIC) images. (C, G, K) Green signals indicate nuclei of OSH1-positive cells visualized by anti-OSH1 antibody and FITC. (D, H, L) Red signals indicate PI-stained nuclei of whole cells. (E, I, M) DIC images. Each set of cross-section images (B–E, F–I, and J–M) is linked to the corresponding part of the longitudinal section (A) by a blue line. White dashed lines in the longitudinal section (A) indicate the borders of the cross sections (B–M). (B, E, F, I, J, M) The circled region in each panel indicates the SAM. (A, B, E, F, I, J, M) White arrowheads indicate P1. Scale bars: 50 μm. (PDF) [file pone.0269374.s001.pdf]

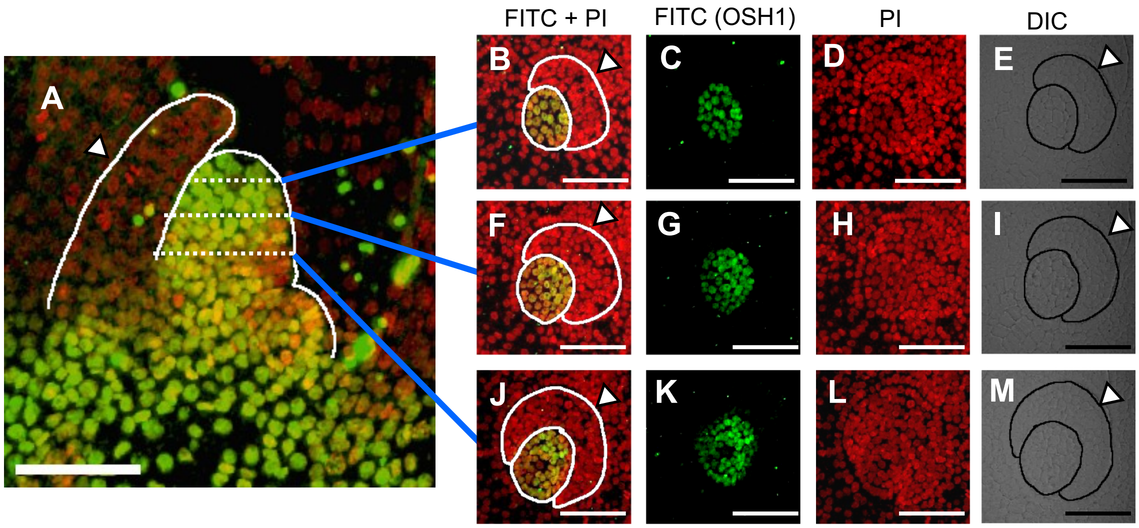

Fig S1. OSH1-immunostained shoot apical meristem of wild type at early P0 stage. (A) A longitudinal section and (B–M) serial cross sections of the wild type SAM at early P0 stage. (B, F, J) Merged images of FITC signals, PI signals, and differential interference-contrast (DIC) images. (C, G, K) Green signals indicate nuclei of OSH1-positive cells visualized by anti-OSH1 antibody and FITC. (D, H, L) Red signals indicate PI-stained nuclei of whole cells. (E, I, M) DIC images. Each set of cross-section images (B–E, F–I, J–M) is linked to the corresponding part of the longitudinal section (A) by a blue line. White dashed lines in the longitudinal section (A) indicate the borders of the cross sections (B–M). (B, E, F, I, J, M) The circled region in each panel indicates the SAM. (A, B, E, F, I, J, M) White arrowheads indicate P1. Scale bars: 50 μm.
