# Supplementary material for: Measurements of the number of specified and unspecified cells in the shoot apical meristem during a plastochron in rice (Oryza sativa) reveal the robustness of cellular specification process in plant development
Source: PLoS One. 2022 Jun 3;17(6):e0269374. doi: 10.1371/journal.pone.0269374 (PMC9165865; doi:10.1371/journal.pone.0269374)
Supplement: S11 Fig — The width of the SAM was defined as the length of the baseline set at the point where P2, which was P1 in the previous stages, was attached to the SAM. The height was defined as the length of a perpendicular line drawn from the peak of the shoot apex to the baseline. The width (yellow line), height (red line), shape (height/width), and volume of the SAM were measured. P1 and P2 indicate leaf primordia. (*) indicates leaf base of P2. (PDF) [file pone.0269374.s011.pdf]

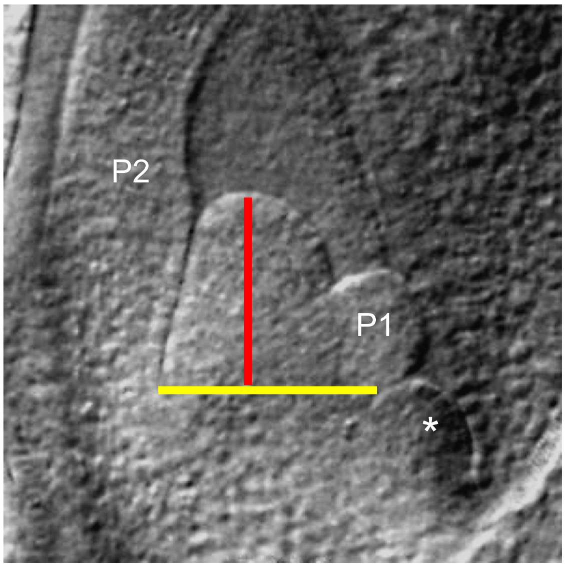

Fig S11. Measurement of size and shape of the shoot apical meristem at the early P1 stage. The width of the SAM was defined as the length of the baseline set at the point where P2, which was P1 in the previous stages, was attached to the SAM. The height was defined as the length of a perpendicular line drawn from the peak of the shoot apex to the baseline. The width (yellow line), height (red line), shape (height/width), and volume of the SAM were measured. P1 and P2 indicate leaf primordia. (*) indicates leaf base of P2.
